# Elevated FBXL18 promotes RPS15A ubiquitination and SMAD3 activation to drive HCC

**DOI:** 10.1097/HC9.0000000000000198

**Published:** 2023-06-28

**Authors:** Hong-Qiang Yu, Feng Li, HaoJun Xiong, Lei Fang, Jie Zhang, Ping Bie, Chuan-Ming Xie

**Affiliations:** 1 Key Laboratory of Hepatobiliary and Pancreatic Surgery, Institute of Hepatobiliary Surgery, Southwest Hospital, Third Military Medical University (Army Medical University), Chongqing, P.R. China; 2 Department of Hepatobiliary Surgery, The Third Affiliated hospital of Chongqing Medical University, Chongqing, P.R. China

## Abstract

F-box and leucine-rich repeat protein 18 (FBXL18) is an E3 ubiquitin ligase that is reported to be involved in the tumorigenesis of various types of cancer. However, it remains unknown whether FBXL18 is correlated with hepatocarcinogenesis.

**Methods and results:**

In the current study, we found that FBXL18 was highly expressed in HCC tissues and positively associated with poor overall survival of HCC patients. FBXL18 was an independent risk factor for HCC patients. We observed that FBXL18 drove HCC in FBXL18 transgenic mice. Mechanistically, FBXL18 promoted the K63-linked ubiquitination of small-subunit ribosomal protein S15A (RPS15A) and enhanced its stability, increasing SMAD family member 3 (SMAD3) levels and translocation to the nucleus and promoting HCC cell proliferation. Moreover, the knockdown of RPS15A or SMAD3 significantly suppressed FBXL18-mediated HCC proliferation. In clinical samples, elevated FBXL18 expression was positively associated with RPS15A expression.

**Conclusion:**

FBXL18 promotes RPS15A ubiquitination and upregulates SMAD3 expression, leading to hepatocellular carcinogenesis, and this study provides a novel therapeutic strategy for HCC treatment by targeting the FBXL18/RPS15A/SMAD3 pathway.

## INTRODUCTION

Hepatocellular carcinoma (HCC), which is the fourth leading cause of cancer-related death worldwide, is the most common primary liver malignancy, accounting for 90% of liver cancer cases^1^. Despite various treatment strategies, including surgical resection, adjuvant chemotherapy and radiotherapy, high-intensity focused ultrasound, and especially the application of molecular-targeted drugs in recent years, the mortality and recurrence rates of HCC remain high. Possible reasons that explain these high mortality and recurrence rates are the robust proliferation and invasive behavior of cancer cells and immune system disorders. Therefore, it is important to identify the precise molecular mechanisms underlying HCC pathogenesis to facilitate the development of novel therapies.

Ubiquitination refers to the attachment of ubiquitin molecules to target proteins through covalent bonds to regulate the stability, activity, and localization of these proteins. E3 ubiquitin-protein ligases, which are the critical components of the ubiquitin-proteasome system, mediate the recognition of specific target proteins^2^. F-box proteins are indispensable substrate-recognition subunits of the SKP1-Cullin1-F-box protein E3 ubiquitin ligase complex and are associated with tumorigenesis and progression^3–6^. F-box proteins can be divided into 3 families, namely, the FBXW, FBXL, and FBXO families, according to their substrate-recognition domain^7^. FBXL family members have an F-box domain and a leucine-rich repeat domain, and they have been reported to be related to the occurrence and development of HCC. As a member of the FBXL subgroup, F-box and leucine-rich repeat protein 18 (FBXL18) regulates apoptosis through the proteasomal degradation of FBXL7 in HeLa cells^8^ and promotes glioma progression by promoting the ubiquitination of AKT^9^. However, the biological function of FBXL18 in HCC and the molecular mechanism have not yet been elucidated.

Small-subunit ribosomal protein S15A (RPS15A) is a member of the ribosomal protein family and is a 40S ribosomal subunit, and it is important for the synthesis of proteins that are related to the expression and transmission of genetic information in humans; abnormal RPS15A expression is associated with various types of cancers^10–12^, including HCC^13^. RPS15A is located at chromosome 16p13.1 and has a full length of 7369 bp, including 5 exons and a 393 bp-long coding domain that encodes a protein consisting of 130 amino acids (MW 14.8 kDa)^13^.

In this study, we found that FBXL18 was upregulated and correlated with poor prognosis in HCC patients. FBXL18 promoted HCC cell proliferation and drove tumorigenesis in FBXL18-overexpressing (OE-FBXL18) mice. Furthermore, FBXL18 interacted with RPS15A and enhanced its stability by ubiquitination; then, SMAD3 was upregulated to promote HCC cell proliferation. Our study suggests that the FBXL18/RPS15A/SMAD3 axis is a novel target for HCC treatment.

## METHODS

### Patient subjects

All the human HCC tissue and paired adjacent normal tissue paraffin specimens were obtained from the Department of Hepatobiliary Surgery, Southwest Hospital, Chongqing, PR China, from 2011 to 2013. Samples of 4% formaldehyde-fixed tissues were used for immunohistochemistry (IHC) and hematoxylin & eosin analysis. This study was approved by the Ethics Committee of Southwest Hospital (KY2020127), and all of the patients provided informed consent. Pathological data and detailed clinical data were obtained from each patient (Table S3, http://links.lww.com/HC9/A333). In addition, the clinical relevance of the mRNA expression of FBXL18 was assessed using a TCGA dataset (downloaded from https://xenabrowser.net) and a GEO dataset (GSE36376).

### Animal studies and the DEN/CCl4-induced animal model of HCC

*FBXL18* allele-overexpressing (OE-FBXL18) mice were generated by Cyagen Biosciences (Guangzhou, China). To generate this mouse strain, fertilized mouse zygotes were co-injected with a mixture of Cas9 mRNA, gRNA targeting the mouse ROSA 26 gene, and a construct carrying mouse FBXL18 complementary DNA (cDNA), and then the zygotes were transferred into pseudopregnant C57BL/6N female mice. PCR and gene sequencing were used to identify mice harboring these constructs. To further enhance liver tumorigenesis, OE-FBXL18 or wild-type (WT) mice were injected with 25 mg/kg DEN (N0756, Sigma–Aldrich, USA) in the abdomen at the age of 2 weeks, followed by 14 weekly injections with 0.5 mL/kg CCl_4_ dissolved in corn oil. At the age of 24 weeks, the mice were sacrificed by isoflurane. The number of tumors was counted, and the largest tumor diameter was measured. Livers were isolated from the mice and photographed. The liver and body weights were measured^14^. Mouse breeding was performed in the specific pathogen-free facility of the Animal Center of Army Medical University. All the animal experiments were approved by the Institutional Animal Care and Use Committee (IACUC) of Army Medical University and followed all the relevant ethical regulations (AMUWEC2020936).

### Cell culture studies

The human HCC Huh7 cell line was obtained from the Fudan Cell Bank (Shanghai, China), and the HepG2 cell line was obtained from ATCC. All the cell lines tested were negative for mycoplasma contamination. All the experiments were carried out in DMEM supplemented with 10% fetal bovine serum (Gibco, New Zealand), 100 U/ml penicillin, and 100 mg/ml streptomycin (Invitrogen, USA) at 37°C, 95% humidity, and 5% CO_2_. For the half-life experiments, Huh7 cells overexpressing FBXL18 were treated with 100 µg/mL cycloheximide (MedChemExpress, USA) at different times.

### Plasmids, siRNA, and lentivirus

The human *FBXL18* gene tagged with 3xFlag was cloned into the pcDNA3.1 + vector for exogenous expression. siRNAs against RPS15A and SMAD3 and a nontargeting siRNA were synthesized by GenePharma (Shanghai, China), and the sequences are shown in supplemental (Table S4, http://links.lww.com/HC9/A333). The lentivirus construct LV-Flag-FBXL18-GFP was purchased from GeneChem (Shanghai, China).

### CCK-8 and colony formation assays

HCC cells were seeded in 96-well plates at 3000 cells per well and incubated for 24 hours; then, the cells were transiently transfected with the indicated plasmids or siRNAs. After 48 hours of incubation, cell proliferation was analyzed using a Cell Counting Kit-8 (CCK-8; Dojindo, Japan) assay. Cell proliferation is presented as relative cell growth compared with the control group (%). For colony formation assays, 1000 treated cells were seeded in 6-well plates in 1.5 mL DMEM supplemented with 1% penicillin and streptomycin and 10% fetal bovine serum. Colonies with >50 cells were counted 14 days later after staining with 0.1% crystal violet staining at room temperature for 30 minutes.

### Cell cycle assay

Huh7 cells were transfected with siRNA control or siRNA targeting FBXL18 for 48 hours. Next, the cells were digested with 0.25% trypsin and then fixed in 70% ethanol overnight. After fixation, the cells were washed twice in a wash buffer. The cells were stained in the dark with 10 μg/mL PI (Beyotime, China) and 200 μg/mL RNase A(Beyotime, China) at final concentrations in PBS for 30 minutes at room temperature and analyzed on a flow cytometer^15^.

### Western blotting analysis

Treated cells were lysed with RIPA lysis buffer (Thermo Fisher Scientific, USA) supplemented with a protease inhibitor mixture (Roche, Switzerland) and then incubated at 4°C for 30 minutes. After incubation, the lysates were centrifuged at 13,000 rpm for 10 minutes at 4°C. After centrifugation, the supernatants were transferred to new Eppendorf tubes. The protein concentrations were quantified using a BCA Protein Assay Reagent Kit (Beyotime, China). Denatured samples were separated by SDS–PAGE (Beyotime, China) and then transferred to NC membranes (GE Healthcare, UK). The following antibodies were used for Western blotting: goat anti-mouse IgG HRP (1:5000) and goat anti-rabbit IgG HRP (1:5000) antibodies were purchased from Cell Signaling Technology (Cell Signaling, Danvers, MA); a rabbit anti-SMAD3 antibody (1:1000) and anti-SMAD3 antibody (1:1000) were purchased from Cell Signaling Technology (Cell Signaling, Danvers, MA); a mouse anti-Flag antibody (1:1000) was purchased from Sigma0–Aldrich (St. Louis, MO); a rabbit anti-RPS15A antibody (1:1000) and anti-SMAD4 antibody (1:1000) were purchased from Abclonal (Wuhan, China); a rabbit anti-β-actin antibody and anti-GAPDH antibody (1:5000) were obtained from Proteintech (Wuhan, China); a rat anti-HA antibody (1:1000) was purchased from Roche (Basel, Switzerland); and a mouse anti-FBLX18 antibody (1:1000) was purchased from Santa Cruz (Santa Cruz, USA).

### Immunohistochemical staining assay

Tissue specimens were fixed in 10% formalin for 72 hours, dehydrated for 5 hours, and embedded in paraffin (Leica, USA). Paraffin-embedded mouse tissue samples were cut into 4 μm-thick tissue sections, and then, the tissue sections were mechanically deparaffinized and incubated in a 2% sodium citrate antigen retrieval solution (Solarbio, USA) at a high temperature and pressure for 2.5 minutes; then, the samples were washed 3 times for 5 minutes each with PBS. Endogenous peroxidase activity was blocked with methanol containing 5% hydrogen peroxide for 10 minutes, and the samples were washed 3 times for 5 minutes each with PBS and incubated with goat serum for 30 minutes at room temperature. Then, the tissue sections were incubated with primary antibodies at 4°C overnight, followed by incubation with a secondary antibody at room temperature for 30 minutes. Next, the tissue sections were incubated with peroxidase at room temperature for 30 minutes. Then, the sections were stained with DAB (ZSGB-BIO, China). Subsequently, the sections were counterstained with hematoxylin (Biosharp, China) for 30 seconds and incubated in warm water for 20 minutes. The tissue sections were dehydrated and preserved using neutral balsam (Biosharp, China) at room temperature. The primary antibodies used were as follows: anti-FBXL18 (Santa Cruz, USA), anti-RPS15A (Abclonal, China), and anti-Ki-67 (Millipore, USA) antibodies. The staining was evaluated by different specialized pathologists who were blinded to the patient characteristics. The staining intensity was determined from 3 different areas for each patient.

### Immunofluorescence assay

Huh7 cells were cultured in 6-well plates with coverslips overnight and then transfected with control and Flag-FBXL18 plasmids for 48 hours. The cells were subsequently fixed with 4% paraformaldehyde for 40 minutes and permeabilized with Triton X-100 for 15 minutes. After blocking for 30 minutes, the cells were incubated with primary antibodies at 4°C overnight, and secondary antibodies were conjugated to fluorophores at room temperature for 1 hour. Finally, the nuclei of the cells were stained with Hoechst 33342. Images of the cells were captured using an Olympus microscope.

### qRT-PCR

Total RNA was isolated from cultured Huh7 and HepG2 cells using RNAiso Plus reagent (TaKaRa, Japan). cDNA was synthesized using the PrimeScript RT Reagent Kit with gDNA Eraser (Perfect Real Time) (TaKaRa, Japan) according to the manufacturer’s instructions. Then, the mRNA levels of the target genes were determined by qRT–PCR using TB Green Premix Ex Taq II (TaKaRa, Japan) with the CFX96 Touch Real-time PCR Detection System. The primers were listed in Table S5, http://links.lww.com/HC9/A333. The results were calculated based on the threshold cycle (Ct), and the relative fold change was determined using the 2^−ΔΔCt^ method.

### Co-immunoprecipitation assay

To examine the interaction between FBXL18 and RPS15A, Huh7 and HepG2 cells were transfected with Flag-FBXL18 plasmids and incubated for 48 hours. The cells were lysed using immunoprecipitation lysate buffer (100 mM NaCl, pH 8.0, 20 mM Tris/HCl, 1% NP-40, and a protease inhibitor cocktail tablet) for 25 min on ice. The lysates were centrifuged at 10,000 × g for 15 minutes at 4°C. After centrifugation, the supernatants were transferred to new Eppendorf tubes, and 1–10 µL (0.2–2 µg) of primary antibody (the optimal antibody concentration was determined by titration) was added and incubated for 1 hour at 4°C. After incubation, 50 µL Protein A/G PLUS-Agarose (Santa Cruz, USA) was added to the protein-antibody complexes and incubated at 4°C on a rotating device overnight. The immunoprecipitates were washed 3 times with immunoprecipitation buffer, and a 5 × sample loading buffer was added to the beads before boiling for 5 minutes. The supernatants were collected and used in a Western blot assay.

### *In vivo* ubiquitination assay

To examine the ubiquitination of RPS15A and the linked form of the polyubiquitin chains by FBXL18, Huh7 cells were transfected with HA-Ub and Flag-FBXL18 plasmids. After 48 hours of transfection, the cells were lysed with immunoprecipitation lysate buffer and denatured by boiling for 5 minutes. The detailed procedures in immunoprecipitation and IB analysis were conducted as described previously. The eluted proteins were analyzed by Western blotting analysis with an anti-RPS15A antibody.

### Proteome analysis

Cell samples were sonicated 3 times on ice using a high-intensity ultrasonic processor in lysis buffer (8 M urea and 1% Protease Inhibitor Cocktail). The remaining debris was removed by centrifugation at 12,000 × g at 4°C for 15 minutes. Then, the supernatants were collected, and the protein concentrations were determined with a BCA Kit according to the manufacturer’s instructions. For digestion, the proteins were reduced by incubation with 5 mM dithiothreitol at 56°C for 30 minutes, followed by alkylation with 11 mM iodoacetamide at room temperature in the dark for 15 minutes. The protein samples were then diluted by adding 100 mM TEAB to achieve final urea concentrations of less than 2 M. Finally, trypsin was added at a 1:50 trypsin-to-protein mass ratio and incubated overnight for the first round of digestion, and a 1:100 trypsin-to-protein mass ratio was incubated 4 hours for the second round of digestion. The samples were subsequently subjected to LC–MS/MS analysis. The tryptic peptides were dissolved in 0.1% formic acid (solvent A) and directly loaded onto a homemade reversed-phase analytical column (15 cm length, 75 μm i.d.). The gradient comprised an increase in solvent B (0.1% formic acid in 98% acetonitrile) from 6% to 23% over 26 minutes, an increase from 23% to 35% in 8 minutes, and a further increase to 80% in 3 minutes; then, the samples were maintained at 80% for the last 3 min. All of these steps were performed all at a constant flow rate of 400 nl/min on an EASY-nLC 1000 UPLC system. The peptides were subjected to an NSI source followed by tandem mass spectrometry (MS/MS) in Q ExactiveTM Plus (Thermo Fisher Scientific) coupled online to the UPLC. The electrospray voltage applied was 2.0 kV. The m/z scan range was 350–1800 for the full scan, and intact peptides were detected in the Orbitrap at a resolution of 70,000. The peptides were then selected for MS/MS using an NCE setting of 28, and the fragments were detected in the Orbitrap at a resolution of 17,500. The data-dependent procedure alternated between 1 MS scan followed by 20 MS/MS scans with 15 sc dynamic exclusion. Automatic gain control was set to 5E4. The fixed first mass was set to 100 m/z. A 1.5-fold change and *p* value < 0.05 were defined as the criteria that indicated statistically significantly different expression in the proteome.

### Data and code availability

All the detailed data in this paper are available upon request. This paper does not report the original code.

### Statistics

Prism 8.0 (GraphPad, USA), SPSS20 (Statistical Product Service Solutions, IBM), R (R 4.1.2), and R-Studio (v7.2 Build 153957) software were used to analyze the data, and the data are presented as the mean ± SEM. Comparisons between 2 groups were analyzed with a 2-tailed unpaired *t*-test. Comparisons among more than 2 groups were analyzed with 1-way ANOVA with Tukey’s post hoc test when the data passed the Shapiro–Wilk normality test. When the data did not pass the normality test, the nonparametric Kruskal–Wallis test was used, followed by Dunn’s multiple range test for post hoc comparisons. Area determination was performed using an imaging system (Olympus, Germany) and FIJI software (ImageJ, National Institutes of Health). The correlations between the genetic expression and clinicopathological factors were performed by Pearson’s chi-square test. OS was analyzed using a log-rank test and the Kaplan–Meier method. The correlation between FBXL18 expression and RPS15A expression was determined by Pearson’s correlation coefficient. *p*≤0.05 were considered statistically significant.

## RESULTS

### FBXL18 is positively correlated with poor survival in HCC patients

**FIGURE 1:**
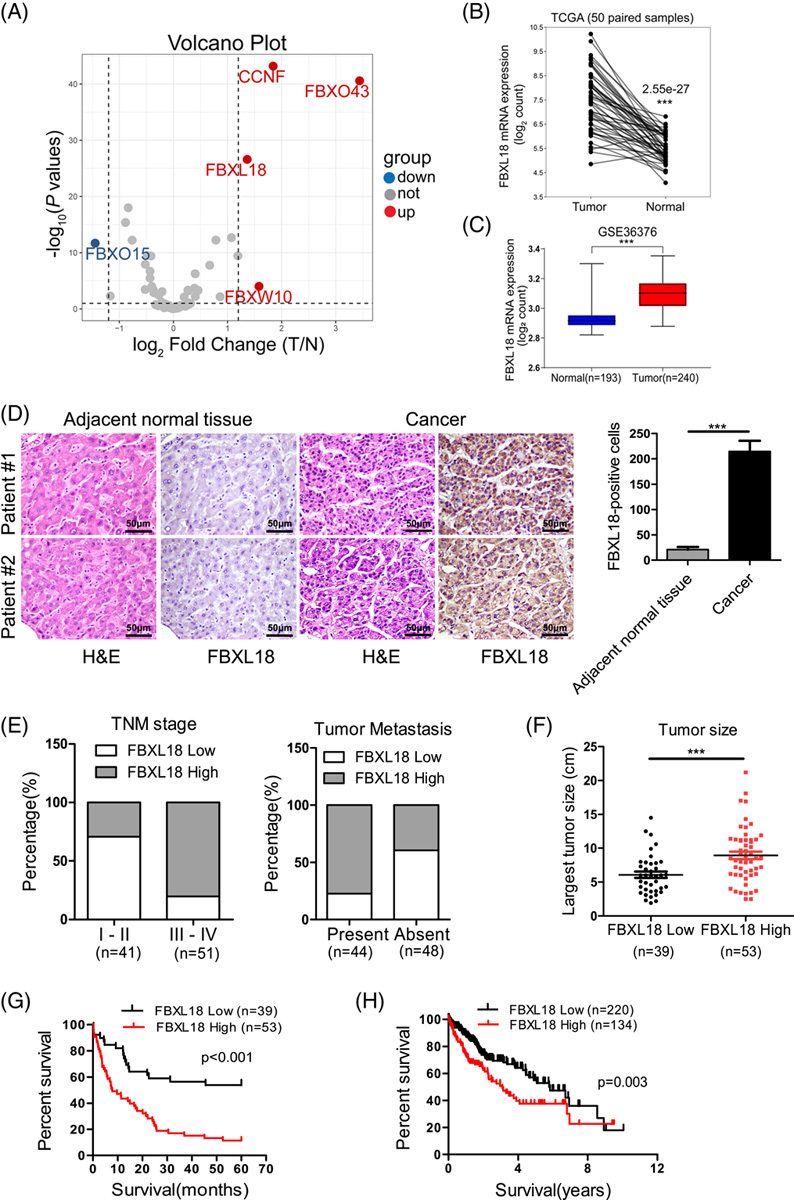
FBXL18 expression is associated with poor survival of HCC patients. (A) The volcano plot shows that the mRNA level of the E3 ubiquitin-protein ligase family was upregulated 1.2-fold in HCC tumors in the TCGA dataset. (B, C) The mRNA level of FBXL18 is more highly expressed in HCC tumors than in normal liver tissues in the TCGA (B) and GSE36376 datasets (C). (D) Tissue sections of HCC and matched adjacent tissues from patients were subjected to HE and IHC staining for FXL18. Representative images are shown. (E, F) The relationships between FBXL18 protein expression and TNM stage and tumor metastasis (E) and tumor size (F) were evaluated. Unpaired Student *t*-test was used. (G, H) The association between FBXL18 expression and overall survival of HCC patients in the Southwest Hospital dataset (G) and TCGA dataset (H) was determined. Overall survival was analyzed through log-rank tests and the Kaplan–Meier method. The data are presented as the mean ± SEM, ***p ≤ 0.001. Abbreviations: FBXL18, F-box and leucine-rich repeat protein 18; IHC, immunohistochemistry; TCGA, The Cancer Genome Atlas.

To determine the role of FBXL18 in human HCC, a volcano plot was generated and showed that the mRNA levels of FBXL18, CCNF, FBXO43, and FBXW10 were significantly highly expressed in HCC tissues according to The Cancer Genome Atlas (TCGA) dataset (Fig. 1A). CCNF, FBXO43 and FBXW10 were reported to be associated with HCC^16–19^, but whether FBXL18 is correlated with HCC remains unknown. The mRNA levels of FBXL18 were significantly higher in HCC tissues than in adjacent normal tissues in the TCGA dataset (Fig. 1B) and in the GSE36376 dataset (Fig. 1C). The protein expression of FBXL18 in a cohort of 92 paired human HCC and adjacent normal liver tissue specimens was measured by IHC staining. The results demonstrated that the expression of FBXL18 was higher in 57.6% (53/92) of the HCC samples than in adjacent normal liver tissues. (Fig. 1D). The relationships between FBXL18 protein expression and clinicopathological parameters were investigated. FBXL18 was positively associated with TNM stage, histological grade, tumor size, vascular thrombosis, and metastasis in HCC patients (Fig. 1E, F and Table 1), indicating that FBXL18 expression is positively correlated with poor prognosis in HCC patients. To extend this analysis, the association between overall survival (OS) and various risk factors in 92 HCC patient tissue samples was analyzed. Kaplan–Meier analysis showed that FBXL18 expression in 92 HCC patients was positively associated with poor survival in HCC patients (*p* < 0.001; Fig. 1G). Consistently, FBXL18 expression in a cohort of 354 HCC patients was positively associated with poor survival in the TCGA dataset (*p* = 0.003; Fig. 1H). Univariate and multivariate analyses revealed that FBXL18 expression (*p* = 0.048), histological grade (*p* = 0.037), and recurrence (*p* = 0.013) were negatively associated with OS in HCC patients, indicating that FBXL18 is an independent risk factor in HCC patients (OS, [HR]: 0.5276; 95% [Cl]: 0.2797–0.9953; *p* = 0.048, Table 2). Consistent with this finding, the TCGA dataset showed that FBXL18 expression was significantly associated with histological grade, recurrence, and fetoprotein (Table S1, http://links.lww.com/HC9/A333) and functioned as an independent risk factor in HCC patients (OS, [HR]: 1.441; [Cl]: 1.009–2.058; *p* = 0.044, Table S2, http://links.lww.com/HC9/A333).

**TABLE 1:** Relationship between FBXL18 and clinicopathological characteristics in the 92 HCC patients in the IHC cohort

Variables	Cases(%)	Low FBXL18	High FBXL18	*p*
Age(y)	—	—	—	0.822
< 55	66 (71.7)	27	39	—
≥ 55	26 (28.3)	12	14	—
Gender	—	—	—	0.798
Female	14 (15.2)	5	9	—
Male	78 (84.8)	34	44	—
TNM Stage	—	—	—	0.001***
I–II	41 (44.6)	29	12	—
III–IV	51 (55.4)	10	41	—
Histologic grade	—	—	—	0.043*
G1G2	75 (81.5)	36	39	—
G3	17 (18.5)	3	14	—
Tumor size	—	—	—	0.02*
≤5 cm	25 (27.2)	16	9	—
>5 cm	67 (72.8)	23	44	—
Recurrence	—	—	—	0.064
Absent	25 (27.2)	15	10	—
Present	67 (72.8)	24	43	—
Vascular thrombosis	—	—	—	0.034*
Absent	66 (71.7)	33	33	—
Present	26 (28.3)	6	20	—
Metastasis	—	—	—	0.001***
Present	44 (47.8)	10	34	—
Absent	48 (52.2)	29	19	—

Abbreviation: FBXL18, F-box and leucine-rich repeat protein 18.

Bold values indicate statistical significance

**TABLE 2:** Univariate and multivariate analyses indicating associations between overall survival and various risk factors in the 92 HCC patients of the IHC cohort

		OS	
Variables	Cases	HR(95% Cl)^#^	*p*
Univariate analysis			
FBXL18(high vs. low)	(53 vs. 39)	0.318(0.1832–0.5521)	<0.001***
Age( ≥ 55y vs. < 55y)	(26 vs. 66)	0.318(0.1832–0.5521)	0.874
Gender(male vs. female)	(78 vs. 14)	1.307(0.6464–2.644)	0.456
Histologic grade(G1G2 vs. G3)	(75 vs. 17)	0.54(0.3024–0.966)	0.038*
TNM Stage(I, II vs. III, IV)	(41 vs. 51)	3.075(1.8–5.255)	<0.001***
Tumor size( > 5 cm vs. ≤ 5 cm)	(67 vs. 25)	2.216(1.182–4.155)	0.013*
Recurrence(present vs. absent)	(67 vs. 25)	2.519(1.271–4.992)	0.008***
Vascular thrombosis(present vs. absent)	(26 vs. 66)	2.672(1.598–4.468)	<0.001***
Metastasis(present vs. absent)	(44 vs. 48)	2.25(1.365–3.708)	0.0015**
Multivariate analysis			
FBXL18(high vs. low)	(53 vs. 39)	0.5276(0.2797–0.9953)	0.048*
Histologic grade(G1G2 vs. G3)	(75 vs. 17)	0.5065(0.2669–0.961)	0.037*
TNM stage(I, II vs. III, IV)	(41 vs. 51)	1.595(0.73–3.4853)	0.241
Tumor size( > 5 cm vs. ≤ 5 cm)	(67 vs. 25)	1.3408(0.6619–2.7162)	0.415
Recurrence(present vs. absent)	(67 vs. 25)	2.5646(1.2177–5.4015)	0.013*
Vascular thrombosis(present vs. absent)	(26 vs. 66)	1.8084(0.8872–3.6861)	0.103
Metastasis(present vs. absent)	(44 vs. 48)	0.6635(0.3039–1.4489)	0.303

Abbreviation: FBXL18, F-box and leucine-rich repeat protein 18.

Bold values indicate statistical significance.

### FBXL18 promotes inflammation and hepatocarcinogenesis in mice

**FIGURE 2:**
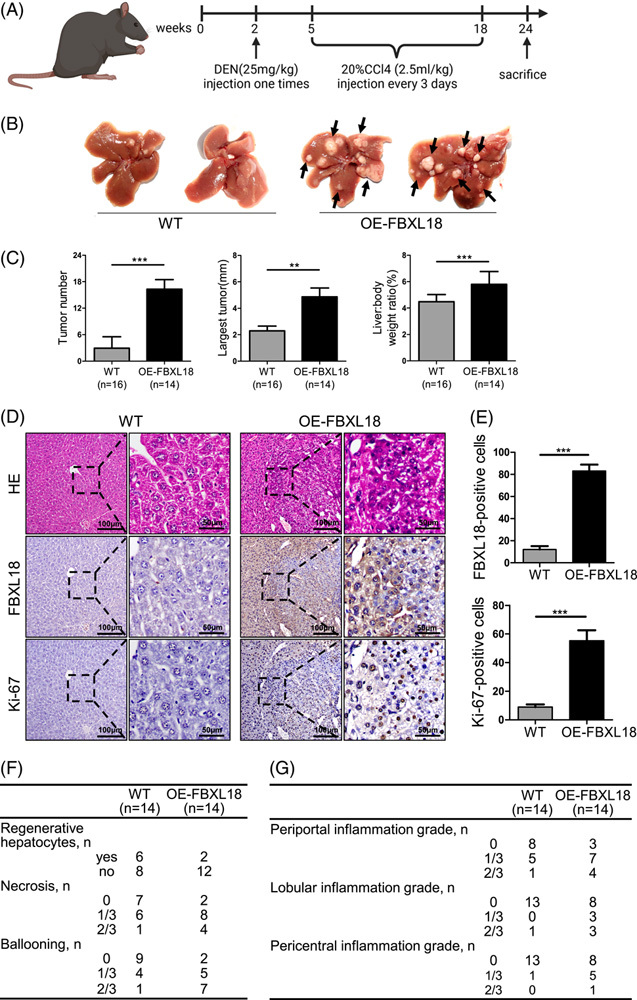
FBXL18 promotes hepatocarcinogenesis and liver inflammation in mice. (A-C) The diagram indicates that OE-FBXL18 (n = 14) and WT (n = 16) mice were injected with DEN at the age of 2 weeks, followed by 14 weeks of i.p. injections with CCl_4_; the mice were sacrificed at 24 weeks of age. Representative images (B), tumor number, liver: body weight ratio and largest tumor size (C) are shown. Arrows indicate tumors; scale bars: 100 or 50 µm. Mice were treated as described in (A). (D, E) H&E and IHC staining assays with the indicated antibodies are shown (D) and analyzed (E), respectively. The data are presented as the mean ± SEM, n = 3. Unpaired Student’s *t-*test was used in C and E. **p ≤ 0.01, ***p ≤ 0.001. (F) OE-FBXL18 mice had severe necrosis and ballooning in the liver compared with wild-type mice. (G) OE-FBXL18 mice had severe inflammation in the liver compared with wild-type mice. Abbreviations: FBXL18, F-box and leucine-rich repeat protein 18; H&E, hematoxylin & eosin; IHC, immunohistochemistry; OE-FBXL18, FBXL18*-*overexpressing; RPS15A, ribosomal protein S15A; WT, wild-type.

Subsequently, the role of FBXL18 in liver tumorigenesis *in vivo* was addressed. In this study, WT mice and *FBXL18-*overexpressing (OE-FBXL18) mice were used and i.p. injected with 25 mg/kg DEN and 2.5 mL/kg 20% CCl_4_ to shorten the observation period. Mice with high *FBXL18* expression (OE-FBXL18 mice; n = 14) developed more liver tumors, larger tumor sizes, and increased liver and body ratios compared with WT mice (n = 16) up until 24 weeks, demonstrating that FBXL18 overexpression drives hepatocarcinogenesis in mice (Fig. 2A-C). Consistently, compared with WT mice, hematoxylin & eosin and IHC staining showed that the expression of FBXL18 and the proliferation biomarker Ki-67 was significantly increased in OE-FBXL18 mice, indicating that FBXL18 overexpression promoted liver tumor growth (Fig. 2D, E). Furthermore, OE-FBXL18 mice tolerated regenerative hepatocytes and were more sensitive to necrosis and ballooning generation than wild-type mice under conditions of DEN/CCl_4_-induced injury (Fig. 2F). In addition, FBXL18 induced more severe mild lobular, periportal and pericentral inflammation in OE-FBXL18 mice than in wild-type mice (Fig. 2G). Taken together, these results indicate that FBXL18 participates in inflammation during the process of HCC tumorigenesis.

### FBXL18 promotes the proliferation of HCC cells

**FIGURE 3:**
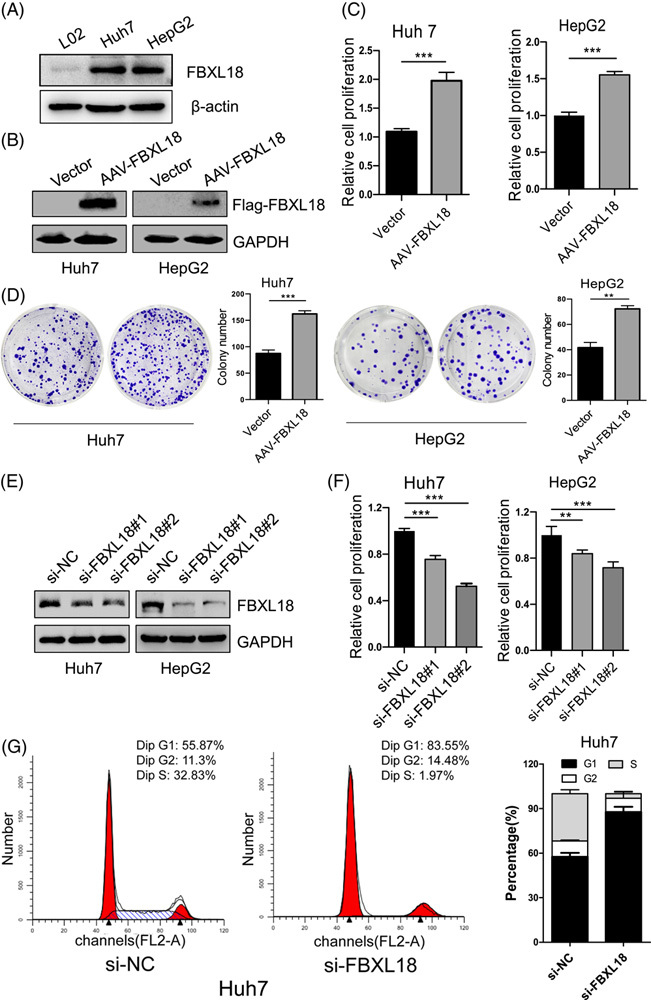
FBXL18 promotes cell proliferation and colony formation in HCC cells. (A) The protein levels of FBXL18 in L02, Huh7, and HepG2 cells were determined by Western blotting. (B, C) HepG2 and Huh7 cells were transfected with Flag-FBXL18 plasmids for 48 hours. The protein levels of FBXL18 and cell proliferation were determined by Western blotting (B) and CCK-8 assays (C), respectively. (D) Colony formation was performed in Huh7 and HepG2 cells after overexpression of FBXL18 for 14 days. (E, F) The effect of FBXL18 knockdown on cell proliferation was evaluated in Huh7 and HepG2 cells after 48 hours knockdown of FBXL18 with siRNAs. The expression of Flag-FBXL18 was detected by Western blotting (E). Cell proliferation was analyzed by CCK-8 assays (F). (G) The cell cycle of Huh7 cells transfected with siRNAs for 48 hours was analyzed by flow cytometry. The data are presented as the mean ± SEM, n = 3. Unpaired Student’s *t*-test was used in C and D. One-way ANOVA with Bonferroni’s multiple comparisons test was used in F. **p ≤ 0.01, ***p ≤ 0.001. Abbreviations: FBXL18, F-box and leucine-rich repeat protein 18; RPS15A, ribosomal protein S15A.

First, we determined the FBXL18 levels in L02, Huh7, and HepG2 cells. The expression of FBXL18 in Huh7 and HepG2 cells was higher than that in L02 cells (Fig. 3A). Next, to evaluate the effect of FBXL18 on HCC cell proliferation, Huh7 and HepG2 cells were transfected with empty vector or Flag-FBXL18 plasmid, and cell proliferation and colony formation assays were then performed. We found that the overexpression of FBXL18 significantly promoted cell proliferation and colony formation in both Huh7 and HepG2 cells (Fig. 3B-D). Reciprocally, silencing of FBXL18 significantly inhibited cell proliferation in Huh7 and HepG2 cells (Fig. 3E, F). Moreover, the cell cycle progression of Huh7 cells transfected with the FBXL18 siRNAs was significantly arrested at the G1 phase, as determined by flow cytometry (Fig. 3G). Taken together, FBXL18 promotes HCC cell proliferation *in vitro*.

### FBXL18 promotes K63-linked RPS15A polyubiquitination and enhances its stability

To identify the direct substrates of FBXL18, Huh7 cells were transfected with Flag-tagged FBXL18, and then pull-down assays were performed with an anti-FBXL18 antibody. The proteins were analyzed by mass spectrometry. A total of 162 proteins were identified among those that were immunoprecipitated with FBXL18.

**FIGURE 4:**
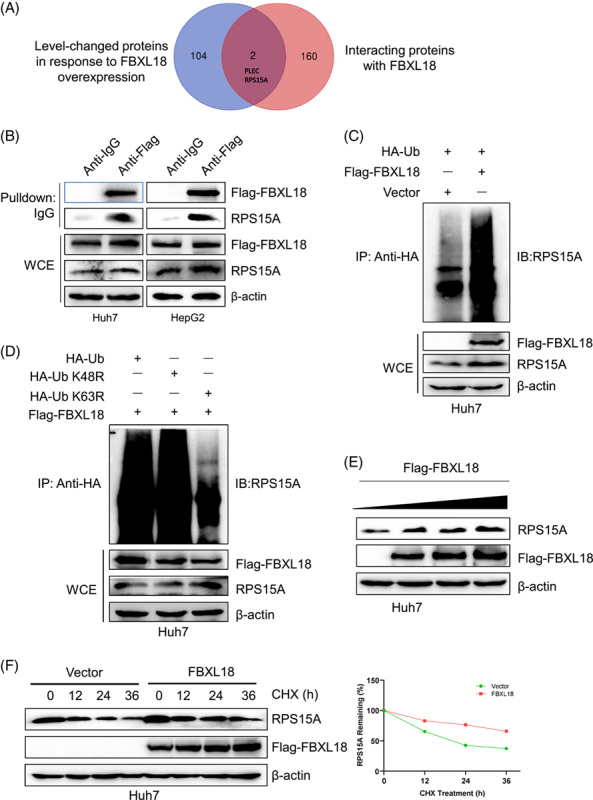
FBXL18 promotes the K63-linked RPS15A ubiquitination and enhances its stability. (A) Venn diagram showing the number of proteins with 1.5-fold changes in expression after FBXL18 overexpression (blue) and FBXL18 interaction candidates identified by co-IP and mass spectrometry (red). (B) FBXL18 bound to endogenous RPS15A. HepG2 and Huh7 cells were transfected with Flag-FBXL18 plasmids for 48 hours and then lysed. The cell lysates were added to the indicated antibodies and protein A/G PLUS-Agarose, followed by Western blotting analysis. WCE, whole cell extract. (C, D) Huh7 cells were transfected with the indicated plasmids and subjected to pull-down using anti-HA antibody or direct Western blotting analysis with the indicated antibodies. (E) Endogenous expression of RPS15A in Huh7 cells after overexpression of FBXL18 for 48 hours. (F) The half-life of RPS15A in Huh7 cells was prolonged by FBXL18 in a cycloheximide chase assay. Cells were treated with 100 µg/mL cycloheximide for different time intervals. Abbreviations: FBXL18, F-box and leucine-rich repeat protein 18; RPS15A, ribosomal protein S15A.

We analyzed all changed proteins and FBXL18-interacting proteins after FBXL18 overexpression using proteome analysis. A total of 106 proteins exhibited a greater than 1.5-fold change after FBXL18 overexpression. Furthermore, 162 proteins were identified as candidates for interaction with FBXL18 in HCC through a pull-down assay using an anti-Flag-FBXL18 antibody. We generated a chart showing the overlapping proteins to identify potential FBXL18 substrates. There were 2 overlapping proteins, PLEC and RPS15A, that exhibited high degrees of change (Fig. 4A). PLEC and RPS15A were reported to be oncogenes in cancers. RPS15A was upregulated and PLEC was downregulated upon FBXL18 expression. RPS15A, but not PLEC, was reported to be associated with HCC^20,21^. These findings indicated that RPS15A may be a key substrate of FBXL18 for HCC tumorigenesis. However, it remains unknown whether the role of RPS15A in HCC depends on its ubiquitination.

To examine the interaction between FBXL18 and RPS15A, Huh7 and HepG2 cells were transfected with Flag-FBXL18, and then, pull-down assays were conducted with an anti-Flag antibody. The results showed that exogenous RPS15A was pulled down by ectopically expressed FBXL18 (Fig. 4B). This finding was further confirmed by FBXL18 LRRs domain binding with RPS15A (Figure S1A, B, http://links.lww.com/HC9/A334). Next, whether FBXL18 promoted RPS15A protein ubiquitination was evaluated. An *in vivo* ubiquitination assay indicated that the overexpression of FBXL18 significantly promoted the polyubiquitination of RPS15A (Fig. 4C). K63-linked ubiquitination promotes protein activation or enhances protein stability, whereas K48-linked ubiquitination mediates protein degradation^6,22,23^. To analyze the type of ubiquitin chains involved in FBXL18-mediated RPS15A polyubiquitination, 2 ubiquitin mutants (K63R and K48R) were used along with wild-type ubiquitin. The ubiquitination assay showed that a K63R ubiquitin mutant significantly reduced the formation of the polyubiquitin chain on RPS15A compared with wild-type ubiquitin and its K48R mutant (Fig. 4D), suggesting that FBXL18 may promote RPS15A stability or activation. We found that FBXL18 upregulated RPS15A expression in a dose-dependent manner (Fig. 4E). Furthermore, FBXL18 prolonged the half-life of RPS15A in a cycloheximide chase assay (Fig. 4F). Taken together, FBXL18 promotes the K63-linked polyubiquitination of RPS15A and enhances its stability.

### FBXL18 promotes cell proliferation via RPS15A-mediated SMAD3 upregulation

**FIGURE 5:**
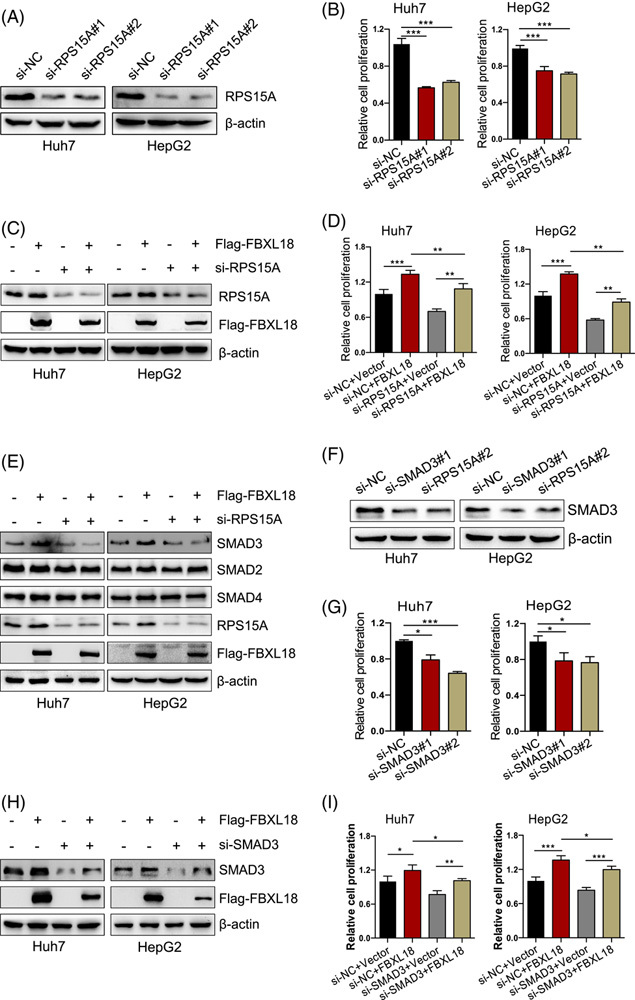
FBXL18 promotes cell proliferation by activating the RPS15A-SMAD3 axis. (A–E) Huh7 and HepG2 cells were transfected siRNA targeting RPS15A (siRPS15A #1 and siRPS15A #2) with or without Flag-FBXL18 for 48 hours. A portion of cells was used to analyze Western blotting with indicated antibodies (A, C, E). The other portion of cells was seeded into 96-well plates after 48 hours for cell proliferation by CCK-8 assay (B, D). (F, G) SMAD3 expression and proliferation were analyzed in cells after silencing of RPS15A or SMAD3 for 48 hours+. (H, I) The effect of SMAD3 knockdown on FBXL18-mediated cell proliferation was analyzed by Western blotting (H) or CCK-8 assay (I). The data are presented as the mean ± SEM, n = 4. One-way ANOVA with Bonferroni’s multiple comparisons test was used in B, D, G, I. *p ≤ 0.05, **p ≤ 0.01, ***p ≤ 0.001. Abbreviations: FBXL18, F-box and leucine-rich repeat protein 18; RPS15A, ribosomal protein S15A.

To validate the effect of RPS15A on FBXL18-mediated HCC cell proliferation, Huh7 and HepG2 cells were transfected with FBXL18 plasmids with or without siRNA targeting RPS15A. The results showed that silencing of RPS15A significantly inhibited HCC cell proliferation, and this effect could significantly block FBXL18-mediated cell proliferation (Fig. 5A-D). The KEGG pathway analysis showed that the SMAD pathway was enriched in FBXL18-overexpressed Huh7 cells (Figure S2, http://links.lww.com/HC9/A334). The SMAD signaling pathway plays a critical role in regulating multiple steps of carcinogenesis. The DNA-binding protein (consisting of SMAD2, SMAD3, and SMAD4) is an important transcription protein that regulates multiple target genes in cooperation with coactivators and corepressors in carcinogenesis. We wanted to determine whether FBXL18-mediated cell proliferation is correlated with SMAD. We found that FBXL18 significantly upregulated the protein expression of SMAD3 but not SMAD2 and SMAD4, and the knockdown of RPS15A blocked this effect (Fig. 5E). Furthermore, knockdown of SMAD3 significantly attenuated FBXL18-mediated cell proliferation in both Huh7 and HepG2 cells (Fig. 5F-I). To validate the effect of SMAD3 on transcription, we determined the SMAD3 levels in Huh7 cells transfected with control or Flag-FBXL18 plasmids by immunofluorescence assay. The results indicate that FBXL18 promoted the translocation of SMAD3 into the nucleus (Figure S3A, B, http://links.lww.com/HC9/A334). Furthermore, we found that FBXL18 upregulated the transcription of c-Myc and HIF-1α^24,25^, the downstream target gene of SMAD3, suggesting FBXL18 enhances SMAD3 transcription-promoting activity (Figure S3C, http://links.lww.com/HC9/A334). Taken together, FBXL18-mediated cell proliferation is attributed to the RPS15A-SMAD3 axis.

### Correlation between FBXL18 expression and RPS15A expression in HCC tissues

**FIGURE 6:**
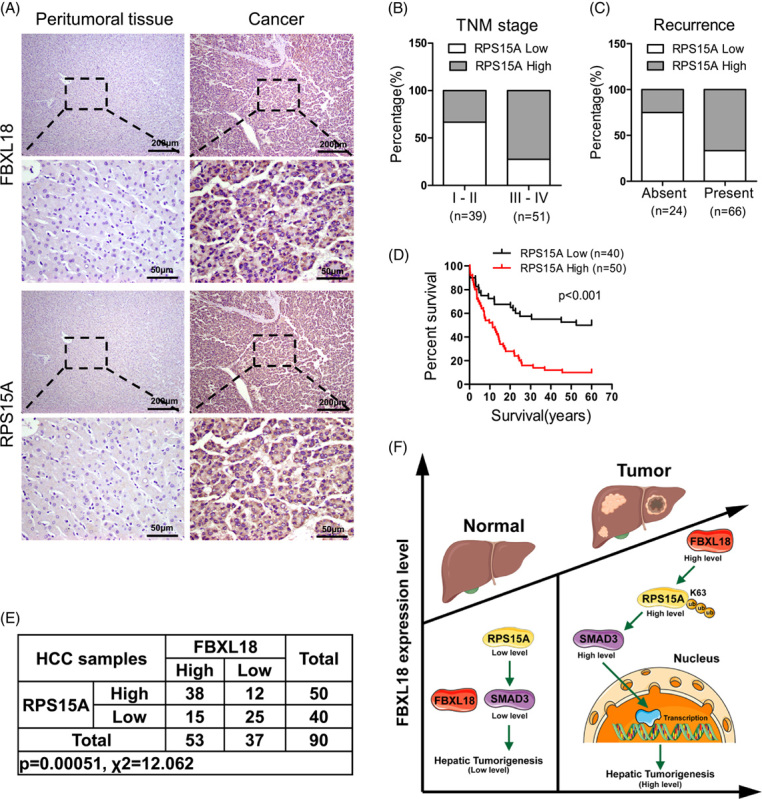
Positive correlation between FBXL18 expression and RPS15A expression in HCC tissues. (A) The expression of RPS15A in HCC tissue samples was determined by IHC staining with an anti-RPS15A antibody. Scale bars: 200 or 50 μm. (B, C) The relationships between the expression of RPS15A and TNM stage and recurrence in HCC patients were determined. (D) RPS15A is positively associated with the overall survival of HCC patients. (E) The association between RPS15A and FBXL18 protein expression in HCC patients was evaluated by Pearson’s correlation coefficient. Calculated using the χ^2^ test. ***p ≤ 0.001. (F) A model of hepatocarcinogenesis triggered by the increased FBXL18 expression through the RPS15A-SMAD3 axis. Abbreviations: FBXL18, F-box and leucine-rich repeat protein 18; IHC, immunohistochemistry; RPS15A, ribosomal protein S15A.

We next evaluated the significance of RPS15A in HCC using patient tissues. First, we used IHC to measure RPS15A expression in 90 paired human HCC tissues and adjacent normal liver tissues. We found that RPS15A was highly expressed in 55.6% (50/90) of HCC tissues (Fig. 6A). Subsequently, the relationship between RPS15A expression and clinicopathological characteristics was evaluated. The expression of RPS15A was positively associated with TNM tumor stage, recurrence, vascular thrombosis, and metastasis (Fig. 6B, C; Table S3, http://links.lww.com/HC9/A333). Furthermore, high RPS15A expression was strongly positively associated with poor OS in HCC patients (*p*<0.001, Fig. 6D). To evaluate the association between FBXL18 and RPS15A, we used IHC to measure FBXL18 expression in HCC tissues and correlated it with RPS15A staining in the same 90 HCC tissues (Fig. 6A). In total, 42.2% (38/90) of HCC tissues had high FBXL18 and high RPS15A staining intensity, which was statistically significant compared to the staining intensity in the normal tissues, suggesting the possibility that FBXL18 stabilized RPS15A in HCC (Fig. 6E). Moreover, the protein levels of RPS15A and SMAD3 were markedly increased in OE-FBXL18 mice (Figure S4, http://links.lww.com/HC9/A334). In conclusion, the increased expression of FBXL18 in HCC patients promoted hepatocarcinogenesis through the induction of RPS15A ubiquitination and upregulation of SMAD3 expression (Fig. 6F).

## DISCUSSION

Due to the lack of understanding of the molecular mechanism underlying tumorigenesis, HCC, with its highly lethal characteristics, is one of the most difficult malignancies to treat. To date, 69 proteins have been identified as members of the F-box family in the human genome^4,26^. Many studies have shown that F-box proteins regulate oncogenes and tumor suppressor genes through polyubiquitination. For example, FBXL16 directly interacts with HIF1α and leads to its ubiquitination-dependent degradation, thus blocking HIF1α-mediated epithelial-mesenchymal transition and angiogenesis in triple-negative breast cancers^27^. Recent studies have revealed that FBXL18 plays crucial roles in the tumorigenesis and prognosis of glioma^9^. Nevertheless, it is still unclear whether FBXL18 accelerates HCC tumorigenesis *in vivo*, and the underlying mechanism is unknown. In this study, we found that FBXL18 protein expression was increased in tumor tissues compared with adjacent normal liver tissues. The abnormally high expression of FBXL18 was correlated with poor survival in HCC patients. Multivariate analysis showed that the FBXL18 protein level was an independent risk factor for HCC patients. Mechanistically, FBXL18 interacted with RPS15A and promoted its stability by polyubiquitination, followed by the upregulation of SMAD3 expression, leading to HCC tumorigenesis *in vitro* and *in vivo*. Collectively, our findings demonstrated the oncogenic role of FBXL18 in HCC and the potential molecular mechanism of the FBXL18-RPS15A-SMAD3 axis in HCC tumorigenesis. Targeting this signaling pathway may be a novel therapeutic strategy for the treatment of HCC patients with high FBXL18 expression.

Compared to other members of the FBXL subgroup, the functions and mechanisms of FBXL18 in cancers remain incompletely understood. FBXL18 was reported to be an oncogene in glioma and cervical cancer. Consistently, we found that FBXL18 drove HCC. A previous study reported that FBXL18 promoted tumorigenesis through the polyubiquitination of AKT and activation of AKT signaling in glioma^9^. FBXL18 could counteract the FBXL7-mediated apoptosis by inducing the ubiquitination-dependent degradation of FBXL7 in cervical cancer^8^. In addition to its role in tumorigenesis, a tumor suppressor role of FBXL18 has been indicated in other cancers^28^. Regarding other functions, FBXL18 targets leucine-rich repeat kinase 2 for degradation by ubiquitination and attenuates the toxicity of neurons, and mutations in FBXL18 are the most common genetic cause of Parkinson’s disease^29^. Here, our data indicated that FBXL18 specifically bound to RPS15A and promoted its polyubiquitination and stability in HCC. Subsequently, RPS15A increased the expression of SMAD3 in Huh7 and HepG2 cells. Here, we reported that FBXL18 promoted hepatocellular carcinogenesis through the activation of the RPS15A-SMAD3 axis.

In conclusion, we provide evidence that FBXL18 is upregulated in HCC tissues and functions as an independent risk factor in HCC patients. Furthermore, FBXL18 plays an important role in promoting the tumorigenesis of HCC by enhancing the polyubiquitination of RPS15A and the subsequent upregulation of SMAD3. Therefore, targeting the FBXL18-RPS15A-SMAD3 axis may be a novel therapeutic strategy for treating HCC patients.

## Supplementary Material

SUPPLEMENTARY MATERIAL
