# Supplementary material for: Elevated FBXL18 promotes RPS15A ubiquitination and SMAD3 activation to drive HCC
Source: Hepatol Commun. 2023 Jun 28;7(7):e00198. doi: 10.1097/HC9.0000000000000198 (PMC10309527; doi:10.1097/HC9.0000000000000198)
Supplement: SUPPLEMENTARY MATERIAL [file hc9-7-e00198-s001.docx]

**Table S1.** Relationship between FBXL18 and clinicopathologic characteristics in the 354 HCC patients of the TCGA database.

| Variables | Cases(%) | Low FBXL18 | High FBXL18 | P Value |
| --- | --- | --- | --- | --- |
| Age(years) |  |  |  | 0.938 |
| ≤ 55 | 118(33.3%) | 73 | 45 |  |
| > 55 | 236(66.7%) | 147 | 89 |  |
| Sex |  |  |  | 0.913 |
| Female | 115(32.5%) | 71 | 44 |  |
| Male | 239(67.5%) | 149 | 90 |  |
| UICC disease stage |  |  |  | 0.074 |
| Ⅰ- Ⅱ | 245(69.2%) | 161 | 84 |  |
| Ⅲ- Ⅳ | 85(24%) | 48 | 37 |  |
| NA | 24(6.8%) | 11 | 13 |  |
| Tumor stage |  |  |  | 0.062 |
| T1/T2 | 261(73.7%) | 169 | 92 |  |
| T3/T4 | 90(25.4%) | 48 | 42 |  |
| NA | 3(0.8%) | 3 | 0 |  |
| Lymph node metastasis |  |  |  | 0.164 |
| N0 | 240(67.8%) | 143 | 97 |  |
| N1 | 3(0.8%) | 1 | 2 |  |
| NX/NA | 111(31.4%) | 76 | 35 |  |
| Cancer distant Metastasis |  |  |  | 0.396 |
| M0 | 256(72%) | 158 | 97 |  |
| M1 | 3(0.8%) | 3 | 0 |  |
| MX | 96(27.2%) | 59 | 37 |  |
| Histologic grade |  |  |  | **0.004**** |
| G1/G2 | 221(62.4%) | 152 | 69 |  |
| G3/G4 | 128(36.2%) | 65 | 63 |  |
| NA | 5(1.4%) | 3 | 2 |  |
| Recurrence |  |  |  | **0.038*** |
| Absent | 136(38.4%) | 93 | 43 |  |
| Present | 168(47.5%) | 103 | 65 |  |
| NA | 50(14.1%) | 24 | 26 |  |
| Vascular invasion |  |  |  | 0.214 |
| Absent | 198(55.9%) | 131 | 67 |  |
| Present | 102(28.8%) | 58 | 44 |  |
| NA | 54(15.3%) | 31 | 23 |  |
| Fetoprotein(ng/ml) |  |  |  | **0.001**** |
| < 20 | 142(40.1%) | 104 | 38 |  |
| ≥ 20 | 126(35.6%) | 65 | 61 |  |
| NA | 86(24.3%) | 51 | 35 |  |

**Table S2.** Univariate and multivariate analyses indicating associations between overall survival and various risk factors in the 354 HCC patients of the TCGA database Supplementary

| Variables | Cases | Univariate analysis OS | |
| --- | --- | --- | --- |
|  |  | Hazard ratio  (95% Cl)^#^ | P value |
| **Univariate analysis** |  |  |  |
| FBXL18(Low vs. High) | (220 vs. 134) | 1.681(1.187-2.379) | **0.003**** |
| Age(> 55y vs. ≤ 55y) | (236 vs. 118) | 1.139(0.785-1.653) | 0.493 |
| Gender(Male vs. Female) | (239 vs. 115) | 0.821(0.574-1.173) | 0.278 |
| Histologic grade(G1/G2 vs. G3/G4 vs. NA) | (221 vs. 128 vs. 5) | 1.16(0.84-1.604) | 0.367 |
| UICC disease stage(Ⅰ/Ⅱ vs. Ⅲ/Ⅳ vs. NA) | (245 vs. 85 vs. 24) | 1.775(1.4-2.25) | **<0.001***** |
| Tumor stage(T1/T2 vs. T3/T4 vs. NA) | (261 vs. 90 vs. 3) | 2.181(1.59-2.991) | **<0.001***** |
| Cancer distant metastasis(M0 vs. M1 vs. MX) | (255 vs. 3 vs. 96) | 1.26(1.046-1.518) | **0.015*** |
| Lymph node stage (N0 vs. N1 vs. NX/NA) | (240 vs. 3 vs. 111) | 1.238(1.03-1.488) | **0.023*** |
| Fetoprotein(< 20 vs. ≥ 20 vs. NA) | (142 vs. 126 vs. 86) | 1.816(1.449-2.275) | **<0.001***** |
| Vascular invasion (Absent vs. Present vs. NA) | (198 vs. 102 vs. 54) | 1.625(1.309-2.017) | **<0.001***** |
| Recurrence(Absent vs. Present vs. NA) | (136 vs. 168 vs. 50) | 6.687(4.954-9.027) | **<0.001***** |
| **Multivariate analysis** |  |  |  |
| FBXL18(Low vs. High) | (220 vs. 134) | 1.441(1.009-2.058) | 0.044* |
| UICC disease stage(Ⅰ/Ⅱ vs. Ⅲ/Ⅳ vs. NA) | (245 vs. 85 vs. 24) | NA | NA |
| Tumor stage(T1/T2 vs. T3/T4 vs. NA) | (261 vs. 90 vs. 3) | 1.566(1.105-2.218) | **0.012*** |
| Cancer distant metastasis(M0 vs. M1 vs. MX) | (255 vs. 3 vs. 96) | 1.22(1.007-1.479) | **0.042*** |
| Lymph node stage (N0 vs. N1 vs. NX/NA) | (240 vs. 3 vs. 111) | NA | NA |
| Fetoprotein(< 20 vs. ≥ 20 vs. NA) | (142 vs. 126 vs. 86) | NA | NA |
| Vascular invasion (Absent vs. Present vs. NA) | (198 vs. 102 vs. 54) | 1.355(1.063-1.728) | **0.014*** |
| Recurrence(Absent vs. Present vs. NA) | (136 vs. 168 vs. 50) | 6.451(4.736-8.787) | **<0.001***** |

**Table S3.** Relationship between RPS15A and clinicopathologic characteristics in the 90 HCC patients of the IHC cohort.

| **Variables** | **Cases(%)** | **Low RPS15A** | **High RPS15A** | **P Value** |
| --- | --- | --- | --- | --- |
| Age(years) |  |  |  | 0.168 |
| < 55 | 64(71.1%) | 25 | 39 |  |
| ≥ 55 | 26(28.9%) | 15 | 11 |  |
| Gender |  |  |  | 0.672 |
| Female | 14(15.6%) | 5 | 9 |  |
| Male | 76(84.4%) | 35 | 41 |  |
| TNM Stage |  |  |  | **0.0004***** |
| I - II | 39(43.3%) | 26 | 13 |  |
| III - IV | 51(56.7%) | 14 | 37 |  |
| Histologic Grade |  |  |  | 0.265 |
| G1G2 | 73(81.1%) | 35 | 38 |  |
| G3 | 17(18.9%) | 5 | 12 |  |
| Tumor Size |  |  |  | 0.108 |
| ≤ 5cm | 25(27.8%) | 15 | 10 |  |
| > 5cm | 65(72.2%) | 25 | 40 |  |
| Recurrence |  |  |  | **0.001**** |
| Absent | 24(26.7%) | 18 | 6 |  |
| Present | 66(73.3%) | 22 | 44 |  |
| Vascular Thrombosis |  |  |  | **0.005**** |
| Absent | 64(71.1%) | 35 | 29 |  |
| Present | 26(28.9%) | 5 | 21 |  |
| Metastasis |  |  |  | **0.001**** |
| Present | 43(47.8%) | 11 | 32 |  |
| Absent | 47(52.2%) | 29 | 18 |  |

**Table S4.** RNA interference sequences

|  | Gene symbol | Forward | Reverse |
| --- | --- | --- | --- |
| Human | FBXL18#1 | CAACCCGUUCUACUUCAGUUU | AAACUGAAGUAGAACGGGUUG |
| Human | FBXL18#2 | CACAGAUCUGAUUCUGAACGU | ACGUUCAGAAUCAGAUCUGUG |
| Human | RPS15A#1 | GCAACUCAAAGACCUGGAATT | UUCCAGGUCUUUGAGUUGCAC |
| Human | RPS15A#2 | AUGGUUACAUUGGCGAAUUTT | AAUUCGCCAAUGUAACCAUGC |
| Human | SMAD3#1 | CUUUGACGAAGCUCAUGCGGA | UCCGCAUGAGCUUCGUCAAAG |
| Human | SMAD3#2 | GAGCCUGGUCAAGAAACUCAA | UUGAGUUUCUUGACCAGGCUC |
| Human | Negative control | UUCUCCGAACGUGUCACGUTT | ACGUGACACGUUCGGAGAATT |

**Table S5.** PCR primer sequences

|  | Gene symbol | Forward | Reverse |
| --- | --- | --- | --- |
| Human | HIF-1α | 5′-GAACGTCGAAAAGAAAAGTCTCG-3’ | 5′-CCTTATCAAGATGCGAACTCACA-3′ |
| Human | c-Myc | 5'-TCAAGAGGCGAACACACAAC-3' | 5'-GGCCTTTTCATTGTTTTCCA-3' |
| Human | β-actin | 5’-CGCGAGAAGATGCCCAGATC-3’ | 5’-TCACCGGAGTCCATCACGA-3’ |
