# Supplementary material for: Elevated FBXL18 promotes RPS15A ubiquitination and SMAD3 activation to drive HCC
Source: Hepatol Commun. 2023 Jun 28;7(7):e00198. doi: 10.1097/HC9.0000000000000198 (PMC10309527; doi:10.1097/HC9.0000000000000198)
Supplement: SUPPLEMENTARY MATERIAL [file hc9-7-e00198-s002.pptx]

## Slide 1
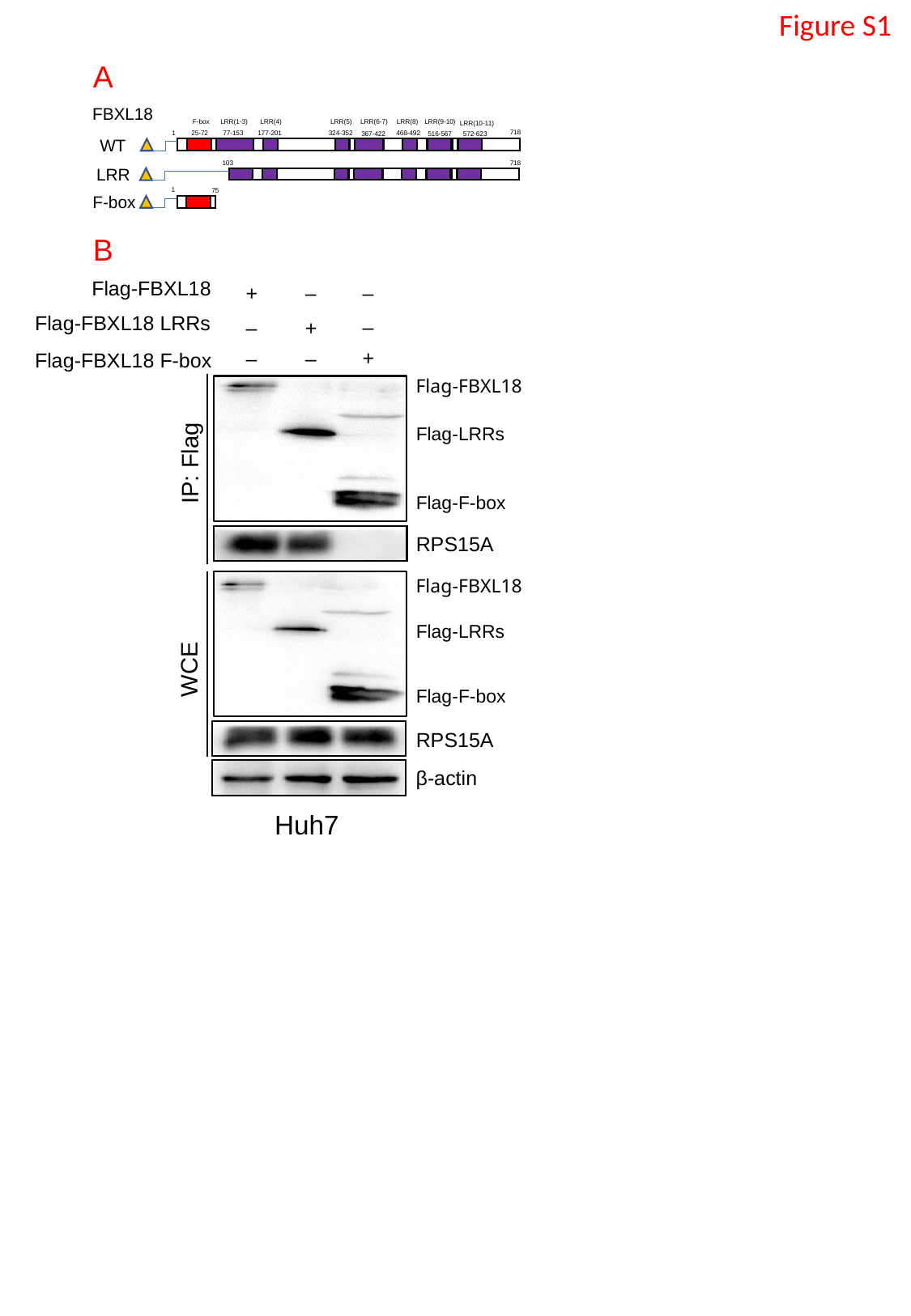

Figure S1
A
FBXL18
F-box
LRR(1-3)
LRR(4)
LRR(5)
LRR(8)
LRR(6-7)
LRR(9-10)
LRR(10-11)
718
1
25-72
77-153
177-201
324-352
468-492
367-422
516-567
572-623
WT
103
718
LRR
1
75
F-box
B
Flag-FBXL18
+
–
–
Flag-FBXL18 LRRs
–
–
+
+
–
–
Flag-FBXL18 F-box
Flag-FBXL18
Flag-LRRs
IP: Flag
Flag-F-box
RPS15A
Flag-FBXL18
Flag-LRRs
WCE
Flag-F-box
RPS15A
β-actin
Huh7

## Slide 2
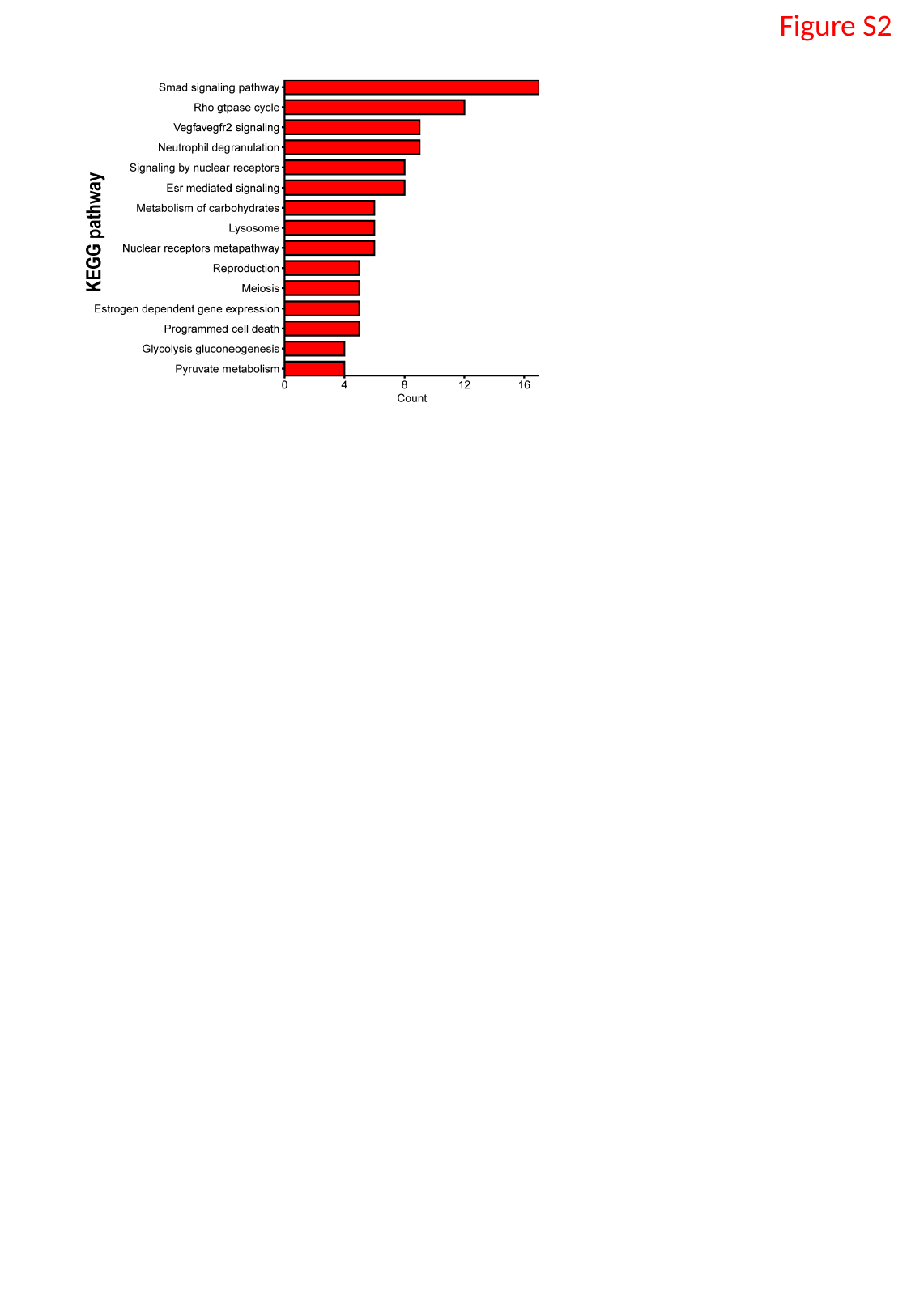

Figure S2

## Slide 3
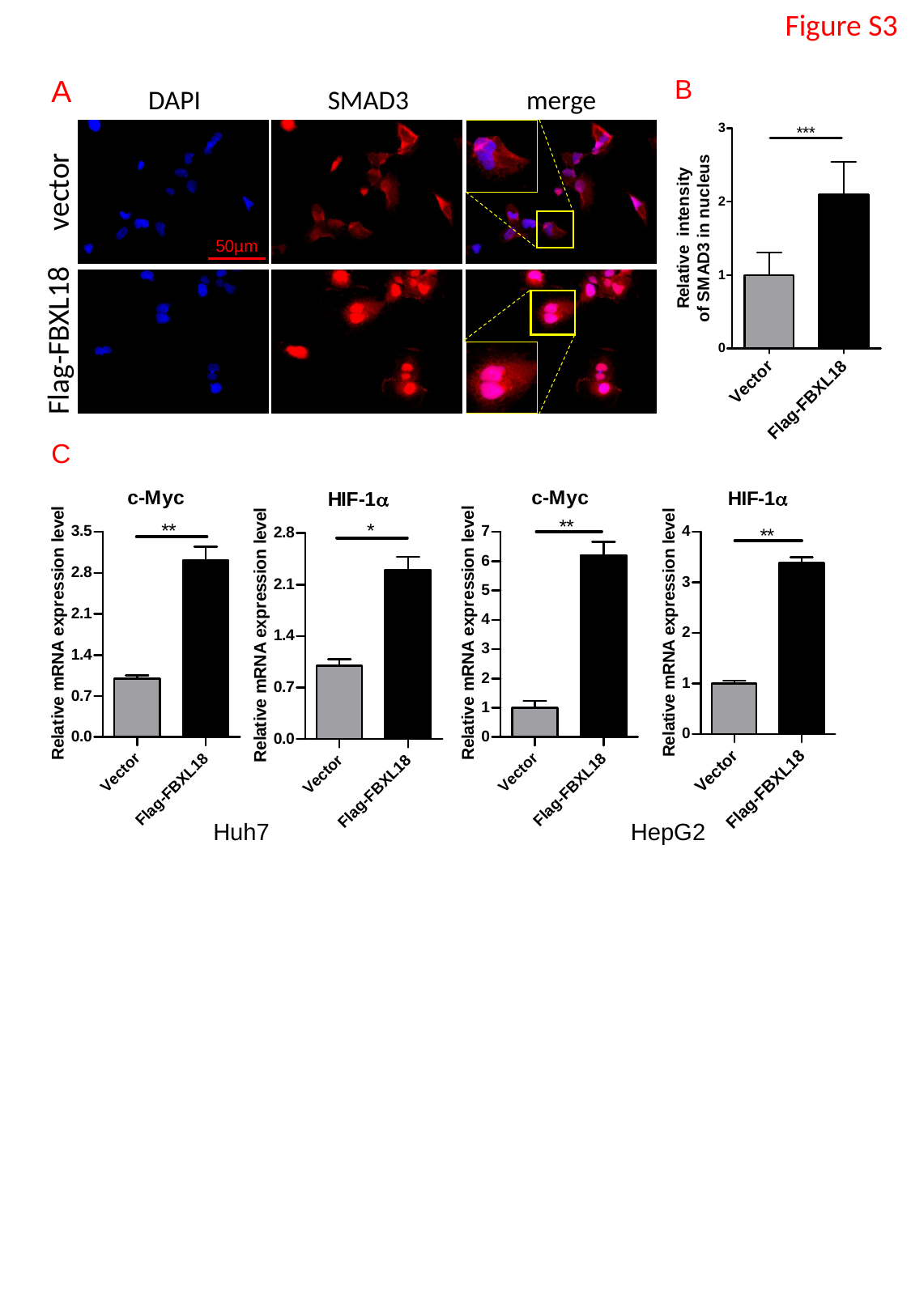

Figure S3
A
B
DAPI
SMAD3
merge
vector
Flag-FBXL18
50µm
C
Huh7
HepG2

## Slide 4
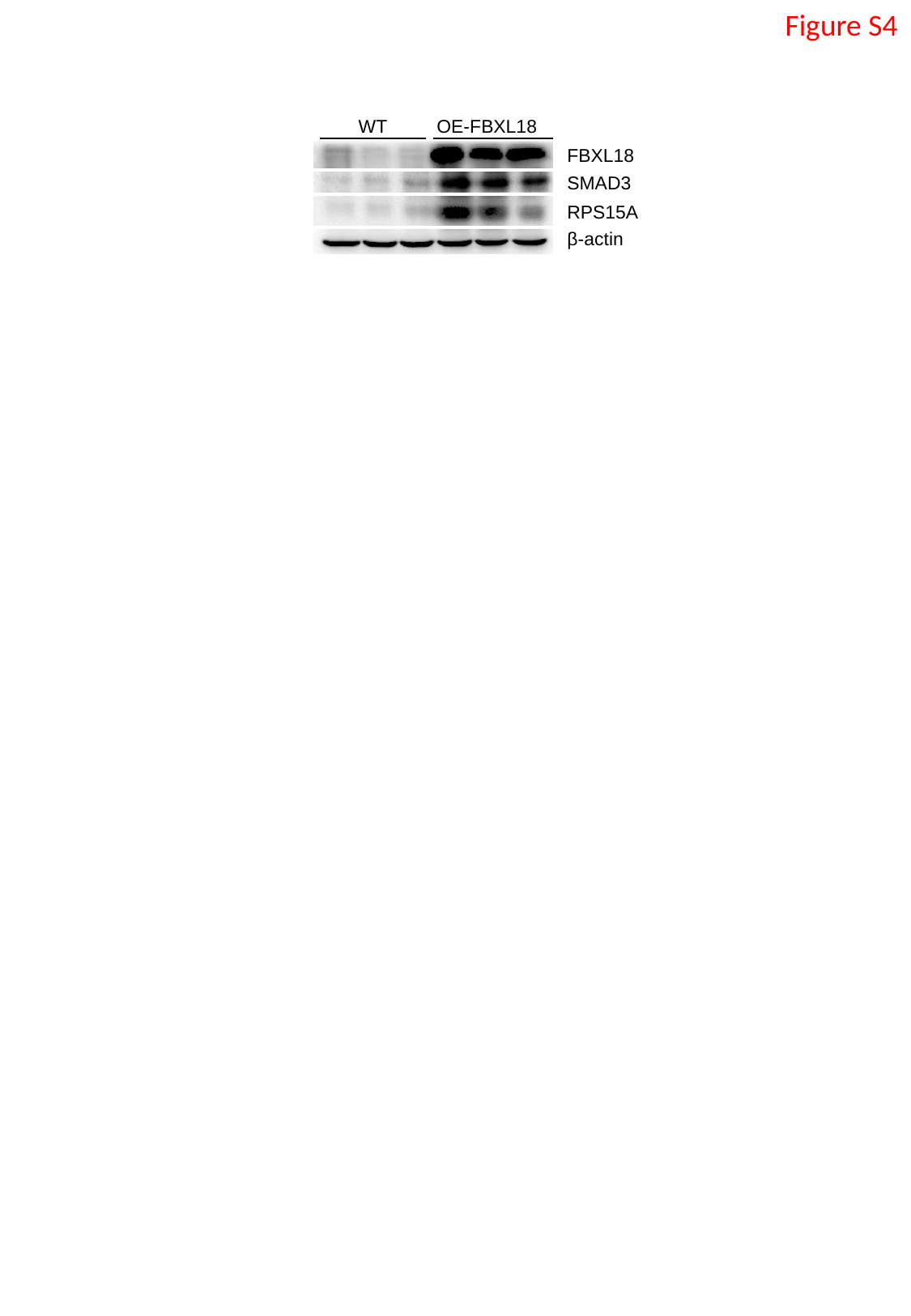

Figure S4
OE-FBXL18
WT
FBXL18
SMAD3
RPS15A
β-actin
